# Altered static and dynamic amplitude of low-frequency fluctuations in acute carbon monoxide poisoning patients: a resting-state fMRI study

**DOI:** 10.3389/fnins.2025.1695556

**Published:** 2026-01-13

**Authors:** Shenghai Wang, Tianze Sun, Wenxuan Han, Xiaoying Lin, Jiaqi Zhang, Yihao Peng, Mingyue Ma, Wei Sheng, Ming Zhang, Haining Li

**Affiliations:** 1 Department of Medical Imaging, The First Affiliated Hospital of Xi'an Jiaotong University, Xi'an, Shaanxi, China; 2 Department of Medical Imaging, Yan'an People's Hospital of Shaanxi Province, Yan'an, Shaanxi, China; 3 Department of Medicine, Xi’an Jiaotong University, Xi’an, Shaanxi, China; 4 MR Research Collaboration, Siemens Healthineers, Shanghai, China; 5 Department of PET/CT, The First Affiliated Hospital of Xi'an Jiaotong University, Xi’an, Shaanxi, China

**Keywords:** carbon monoxide poisoning, cognitive function, dynamic amplitude of low-frequency fluctuation, intrinsic brain activity, resting-state functional magnetic resonance imaging

## Abstract

**Objective:**

This study aimed to investigate alterations in brain activity due to acute carbon monoxide poisoning (ACOP) and their relationship with clinical manifestations using static and dynamic amplitude of low-frequency fluctuation (ALFF) analyses.

**Methods:**

Resting-state functional magnetic resonance imaging (fMRI) and clinical data were obtained from 31 ACOP patients and 28 healthy controls. The static ALFF value and dynamic ALFF variability were measured and compared between groups. Partial correlation analysis explored the relationships between changes in ALFF and clinical features in ACOP patients.

**Results:**

ACOP patients exhibited increased dynamic ALFF in the bilateral superior frontal gyrus (SFG) and left middle frontal gyrus (MFG) and decreased static ALFF in the left middle occipital gyrus (MOG) compared to controls. Aberrant dynamic ALFF in the left SFG and MFG was negatively correlated with MoCA-B scores (*r* = −0.430, *p* = 0.036; *r* = −0.439, *p* = 0.032).

**Conclusion:**

ACOP patients exhibited abnormal instability in intrinsic brain activity, particularly in prefrontal areas, where temporal variability in local brain activity correlates with cognitive performance. This study highlights the value of combined static and dynamic ALFF approaches in understanding brain disturbances caused by CO exposure, providing new insights into the neuropathological mechanisms of ACOP.

## Introduction

Carbon monoxide (CO) is the most prevalent gas poisoning globally. The worldwide cumulative incidence of CO poisoning is 37 cases per million people, with a mortality rate of 4.6 deaths per million (Mattiuzzi and Lippi, 2020). Although the mortality rate and patient death percentage have significantly decreased in many countries, CO poisoning remains a grave concern in regions where wood and other carbon-containing fuels serve as primary domestic energy sources.

In acute CO poisoning (ACOP), CO competitively inhibits oxygen binding to hemoglobin, resulting in the formation of carboxyhemoglobin (COHb), which impairs the transportation of oxygen. Furthermore, CO can attach to additional heme-bearing proteins, such as myoglobin found in cardiac and skeletal muscles, as well as mitochondrial cytochrome c oxidase, thereby intensifying the effects of hypoxia (Rose et al., 2017). Organs that require the most oxygen, including the brain and the heart, are particularly susceptible to damage. Patients who suffer from CO poisoning often experience memory impairment, cognitive dysfunction, anxiety, depression, and motor/vestibular deficits (Ning et al., 2020) during rehabilitation. Severe CO poisoning can rapidly escalate cognitive dysfunction as hypoxia worsens (Weaver, 2009).

In case of CO poisoning, conventional imaging technologies that focus on brain structures often have difficulty evaluating the condition or predicting prognosis (Beppu, 2013). Structural imaging of ACOP can reveal focal cortical injuries, basal ganglia lesions (particularly in the globus pallidus), diffuse atrophy, and white matter demyelination (Lo et al., 2007). While structural imaging sheds light on specific anatomical changes in ACOP, such as basal ganglia lesions, the underlying functional disruptions may not be fully revealed. Recently, functional magnetic resonance imaging (fMRI), an innovative non-invasive approach for assessing spontaneous brain activity, has emerged as a pivotal tool in brain injury studies (Medaglia, 2017). By employing fMRI, researchers have gained insights into specific brain regions affected by CO exposure. For instance, one study simulating the CO intake of smokers found that even low-dose CO inhalation could alter the blood oxygenation level-dependent (BOLD) response in individuals who had never smoked (Bendell et al., 2020), highlighting the nuanced effects of CO on brain functions. Furthermore, two distinct studies, one focusing on acute CO poisoning (Dinghua et al., 2013) and the other on delayed encephalopathy after acute carbon monoxide poisoning (DEACMP) (Wu et al., 2020), utilized the regional homogeneity (ReHo) method and revealed significant alterations in ReHo across various brain regions, such as the bilateral superior frontal gyrus, bilateral basal ganglia, and right insula. While these studies enhance our understanding of CO poisoning, they often assume that local brain activity is static during fMRI scanning, overlooking the dynamic nature of local brain activity.

A growing body of evidence suggests that brain activity fluctuates and remains dynamic, even in resting states (Allen et al., 2014; Park et al., 2018; Liao et al., 2019). By integrating the amplitude of low-frequency fluctuation (ALFF) with “sliding-window” techniques, researchers have introduced the dynamic ALFF method to capture the temporal shifts in ALFF (Fu et al., 2018). Research has shown that ALFF is a reliable parameter for assessing localized intrinsic brain activity (Zang et al., 2007). Furthermore, the ALFF is potentially correlated with glucose metabolism (Aiello et al., 2015) and structural morphology (Liao et al., 2016). This innovative dynamic ALFF approach offers insights into fluctuating local brain functions (Liao et al., 2019). Compared to conventional resting-state measures, such as static ALFF, ReHo, or static functional connectivity, dynamic ALFF is particularly sensitive to short-term variability in regional spontaneous activity and may therefore better capture transient instability in neural systems exposed to fluctuating hypoxic–ischemic injury, as in patients with ACOP. Furthermore, in contrast to task-based fMRI or positron emission tomography, dynamic ALFF can be derived from a brief resting-state scan without task demands or ionizing radiation, which is advantageous for patients in the acute phase. In the context of patient studies, the dynamic ALFF method has been utilized in patients with chronic insomnia (Chen et al., 2022), generalized anxiety disorder (Cui et al., 2020), and minimal hepatic encephalopathy (Guo et al., 2022). Nevertheless, whether patients with CO poisoning exhibit aberrant local dynamic brain activity remains to be determined. Identifying these irregularities could provide insights into the neural underpinnings of CO poisoning.

In this study, we aimed to investigate the differences in the static and dynamic states of local intrinsic brain activity between patients with CO poisoning and healthy controls (HC) using both dynamic ALFF and static ALFF techniques. Additionally, we examined the correlation between atypical local intrinsic brain activity and the clinical manifestations in patients with CO poisoning.

## Materials and methods

### Participants

This study was conducted at Yan’an People’s Hospital from May 2021 to January 2023, enrolling patients with ACOP admitted to the emergency department. Inclusion criteria included a clear history of exposure to coal combustion-derived carbon monoxide, with initial carboxyhemoglobin (COHb) levels exceeding 5% in non-smokers and 10% in smokers upon arrival at the emergency room (Hampson et al., 2012), alongside manifestations of acute central nervous system damage. CO emissions in residential areas often result from poor ventilation or malfunctioning coal stoves, which serve as primary winter heating sources in some northwestern Chinese households. A total of 31 ACOP patients aged 18–75 years were recruited. All were right-handed, with no complications from drug overdose or alcohol/other psychoactive substance poisoning. One out of the 31 patients was a smoker. Each patient underwent an MRI within a maximum of 7 days (average 3.5 ± 2.3 days) post-poisoning. Exclusion criteria are as follows: (a) no history of neurological illness or neuropsychiatric conditions; (b) no family history of demyelinating diseases or Parkinsonism; (c) no history of stroke, encephalitis, or other intracranial diseases; (d) no history of cranial trauma or surgery; and (e) no traumatic injury or subacute carbon monoxide poisoning found on clinical examination.

In this study, 28 healthy controls (HC), who were matched to the patient group based on age, sex, and education level, were included. Inclusion criteria for HC are as follows: (a) no history of carbon monoxide, alcohol, or pesticide poisoning; (b) no history of intracranial lesions, surgeries, or strokes; (c) no history of psychiatric or neurological disorders; and (d) no contraindications for MRI scanning.

The study was approved by the ethics committee of Yan’an People’s Hospital. All participants provided written informed consent before any study-related procedures.

### Clinical and neuropsychological assessments

We collected demographic details, causes and duration of CO exposure, time from the end of exposure to MRI, initial Glasgow Coma Scale (GCS) score, pre-MRI treatment methods, duration of unconsciousness, and laboratory findings, including COHb, from the electronic clinical management system. To assess cognitive function within 24 h of MRI, the Chinese Montreal Cognitive Assessment Basic (MoCA-B) was used. It is effective for evaluating mild cognitive impairment (MCI), particularly in visual space, executive function, and delayed recall (Hoops et al., 2009). Cognitive dysfunction was defined as a MoCA-B score below 26. Based on the educational background, those with less than 12 years of schooling received an extra point on their final MoCA-B score.

### Medical management

All patients received oxygen therapy via face masks. Possible treatments included vasopressor administration and symptomatic relief as needed. Hyperbaric oxygen therapy (HBOT) was indicated for severe poisoning symptoms (e.g., unconsciousness, neurological manifestations, cardiovascular impairment, or significant acidosis) or COHb levels ≥ 25%. All participants received HBOT before the MRI. HBOT was scheduled twice daily (2.5 atmospheres absolute, 115 min, with a 4-h interval) for 3 days and for then once daily (2.2 ATA, 115 min). During hospitalization, HBOT was given 20 times.

### MRI acquisition

A 3.0 Tesla MR scanner (Magnetom Prisma, Siemens Healthineers, Germany) with a 20-channel head coil was used to acquire the brain MRI data. Every participant was instructed to close their eyes, during which time foam cushions and earplugs were employed to minimize the possibility of head movement. Resting-state fMRI data were obtained using a single-shot echo-planar imaging sequence. The scan parameters were as follows: repetition time (TR) = 2,000 ms, echo time (TE) = 30 ms, flip angle = 76°, number of slices = 40 slices, matrix = 64 × 64, field of view (FOV) = 200 mm × 200 mm, voxel size = 3.5 mm × 3.5 mm × 3.5 mm, slice thickness was maintained at 3.5 mm, and there was no gap between slices. The acquisition consisted of 210 measurements and lasted 428 s. High-resolution 3D T1-weighted anatomical images were acquired using a magnetization-prepared rapid gradient-echo (MPRAGE) sequence. The scan’s parameters were as follows: TR = 2,300 ms, TE = 2.26 ms, flip angle = 8°, matrix = 256 × 256, FOV = 256 mm × 256 mm, voxel size = 1.0 mm × 1.0 mm × 1.0 mm. Additionally, the slice thickness was maintained at 1 mm with no gap between the slices, and 192 slices were recorded.

### Data preprocessing

To process the structural and functional MR images, we employed DPABISurf_V1.7 software^1^ and fMRIPrep 20.2.5 toolkit^2^ (Esteban et al., 2019). Notably, fMRIPrep is grounded in the Nipype 1.6.1 framework^3^ (Gorgolewski et al., 2011).

### Anatomical data preprocessing

To correct the non-uniformity of intensity in the T1-weighted image, the N4BiasFieldCorrection (Tustison et al., 2010) tool from ANTs 2.3.3 (Avants et al., 2008) was used. Throughout the workflow, this image served as a T1-weighted reference. Subsequently, the T1-weighted reference image underwent skull stripping using the Nipype version of the antsBrainExtraction.sh method, which is based on ANTs. The OASIS30ANTs template was selected as the optimal choice for this procedure. The brain-extracted T1-weighted image was used to segment into cerebrospinal fluid (CSF), white matter (WM), and gray matter (GM) using fast (FSL 5.0.9). Afterwards, the T1-weighted reference underwent skull stripping using a Nipype modification of the antsBrainExtraction.sh workflow from ANTs, selecting OASIS30ANTs as the desired template. The brain-extracted T1-weighted images were segmented into brain tissue, CSF, WM, and GM using the fast method (FSL 5.0.9) (Zhang et al., 2001). Recon-all from FreeSurfer 6.0.1 was utilized to reconstruct the brain surfaces (Dale et al., 1999). The previously estimated brain mask was improved using a modified version of the technique to align the cortical gray matter segmentation derived from Mindboggle (Klein et al., 2017) with that derived from ANTs and FreeSurfer. ANTs were employed for non-linear registration to achieve spatial normalization based on volume to standard spaces. This process entailed the use of brain-extracted forms of both the T1-weighted reference and corresponding template. ICBM 152 non-linear symmetrical template (version 2009) (Fonov et al., 2011) was selected for normalization.

### Functional data preprocessing

Preprocessing was performed on the functional data of each participant. To ensure the stability of the gradient and tissue excitation levels, the first 10 functional volumes were initially excluded. By employing the unique features of fMRIPrep, a volume serving as a reference, along with its skull-stripped counterpart, was generated. Alignment of the BOLD reference with the T1-weighted reference was achieved using FreeSurfer’s bbregister by employing a boundary-based registration technique (Greve and Fischl, 2009). Slice-timing corrections on the BOLD runs were executed with a 3dTshift from the AFNI (Cox and Hyde, 1997). Head-motion parameters in relation to the BOLD reference were determined using MCFLIRT, a feature of the FSL (Jenkinson et al., 2002). Subsequently, the data were resampled onto the MNI152NLin2009cAsym space for volumetric analysis. To minimize the smoothing effects of the kernel, ants apply transforms from the ANTs were used for volumetric resampling with Lanczos interpolation. To mitigate the influence of head movement on outcomes, the Friston 24-parameter model (Friston et al., 1996) was employed to eliminate the effects of head motion. Subsequently, regression techniques were employed to remove undesirable variables, including signal intensities originating from white matter and cerebrospinal fluid. Afterwards, the linear trends were excluded. Ultimately, the linear trend in fMRI data was eliminated. For more details regarding the pipeline, please consult the workflow section of the fMRIPrep documentation.

Before commencing each analysis, the raw and preprocessed data were visually examined to identify any image artifacts and motion. To minimize the effects of head movement on the processing of subsequent data, we established the following data quality control criteria: Participants who experienced a displacement greater than 3 mm in any axial plane (*x*, *y*, or *z*) or a rotation exceeding 3° during the scanning process were excluded from the study. Next, the average framewise displacement (FD) (Jenkinson et al., 2002) was calculated to assess the head movement. An FD threshold of 0.25 mm was set, and any participants with a value greater than the threshold were excluded. Following completion of quality control, a total of 28 patients with ACOP and 26 HC were included in the subsequent analysis after excluding five subjects (three ACOP patients and two HC).

### Static and dynamic ALFF calculation

DPABI software (version 6.1) was used to calculate the static and dynamic ALFF measures. DPABI is a tool for processing and analyzing brain imaging data that can be found at http://rfmri.org/dpabi (Yan et al., 2016). Initially, the time series for a specific voxel is extracted. Afterwards, using the Fourier transform, the magnitude of all frequencies within a specified range (0.01–0.1 Hz for this particular investigation) was calculated. This was then transformed into a power spectrum to ascertain the measured static ALFF values. Subsequently, the static ALFF values for each voxel were converted to z-scores. This procedure was performed by subtracting the mean and dividing it by the standard deviation of all values.

The sliding window method was employed to calculate the dynamic ALFF. The window length is a crucial yet open parameter in resting-state dynamic computations. To avoid false fluctuations, the length of the window should be greater than 1/*f*_min_, where *f*_min_ represents the minimum frequency of the time series (Leonardi and Van De Ville, 2015). As a result, we decided to use a sliding window size of 50 TRs and a step size of 1 TR to calculate the dynamic ALFF of each participant, which aligns with prior research (Cui et al., 2020; Liu et al., 2021). The window type was selected as Hamming. For each participant, the time series was segmented into 151 windows, and an ALFF map was calculated for every individual window. To assess the temporal variability of the ALFF, referred to as the dynamic ALFF, the standard deviation (SD) of the ALFF for each voxel across 151 windows was calculated. Finally, the static and dynamic ALFF maps underwent spatial smoothing using an isotropic Gaussian kernel, with a full width at half maximum of 6 mm.

### Statistical analysis

We utilized the Mann–Whitney *U* test in SPSS 26.0 (IBM SPSS 26.0, SPSS, Inc., USA) to assess age, education, neuropsychological assessments, and mean FD across the two groups. To identify differences in sex distribution, a chi-squared test was employed, with a significance level of *p* < 0.05. To investigate the distribution patterns of static ALFF and dynamic ALFF variability within each group, the average values of static ALFF and dynamic ALFF variability were calculated for each voxel across individuals in both the HC and ACOP groups. To compare whole-brain static and dynamic ALFF, a two-sample *t*-test was used, with age, sex, education level, and mean FD as covariates. To address multiple comparisons, the results were corrected using the Gaussian random field (GRF) theory. This involved setting a voxel-level threshold of *p* < 0.001 and a cluster-level threshold of *p* < 0.05, both using a two-tailed approach. Partial correlation assessments were performed to explore the relationships between variations in abnormal static or dynamic ALFF and clinical parameters, as well as neuropsychological scales. In these analyses, factors such as age, sex, and educational level were considered as covariates. The *p*-value was set at a significance level of 0.05, indicating statistical significance.

### Validation analysis

To enhance the precision and consistency of our findings, we experimented with different window lengths (30TR, 70TR) and a step size of 4TR for intergroup comparisons.

## Results

### Demographic indices and clinical scales

Table 1 shows the initial demographic characteristics of both patient and HC groups. There was no statistically significant difference between the ACOP and HC groups in terms of sex, age, or educational attainment (*p* > 0.05). The mean FD was computed for both groups, and no significant difference in the mean FD was observed between the groups (*p* > 0.05). There was a significant difference in the MoCA-B scores between the ACOP and HC groups (*p* < 0.001).

**Table 1:** Demographic and clinical characteristics of patients with ACOP and HC.

Variables	Patients (*n* = 28)	HC (*n* = 26)	*χ*^2^/*U*	*p*-value
Demographic data				
Gender (male/female)	8/20	12/14	1.787^a^	0.181
Age (years)	57.0 (28.0)	57.5 (18.0)	-0.407^b^	0.684
Education (years)	12 (3)	12 (0)	-1.172^b^	0.241
Clinical scales				
CO exposure time (h)	6.0 (6.0)	–	–	–
Coma time (h)	2.0 (6.0)	–	–	–
GCS	8.5 ± 3.4	–	–	–
Carboxyhemoglobin (%)	20.1 ± 10.8			
MoCA-B	21.0 (7.0)	29 (2.0)	−6.135^b^	<0.001^*^
Head motion parameter				
Mean FD	0.08 (0.06)	0.09 (0.07)	−0.017^b^	0.986

Data are presented as the median (interquartile range) or mean ± standard deviation. ACOP, acute carbon monoxide poisoning; HC, healthy controls; CO, carbon monoxide; GCS, Glasgow Coma Scale; MoCA-B, Montreal Cognitive Assessment Basic; FD, framewise displacement. ^a^Chi-square test. ^b^Mann–Whitney *U* test. ^*^p < 0.05.

### Changes of ALFF properties in ACOP patients

**Figure 1:**
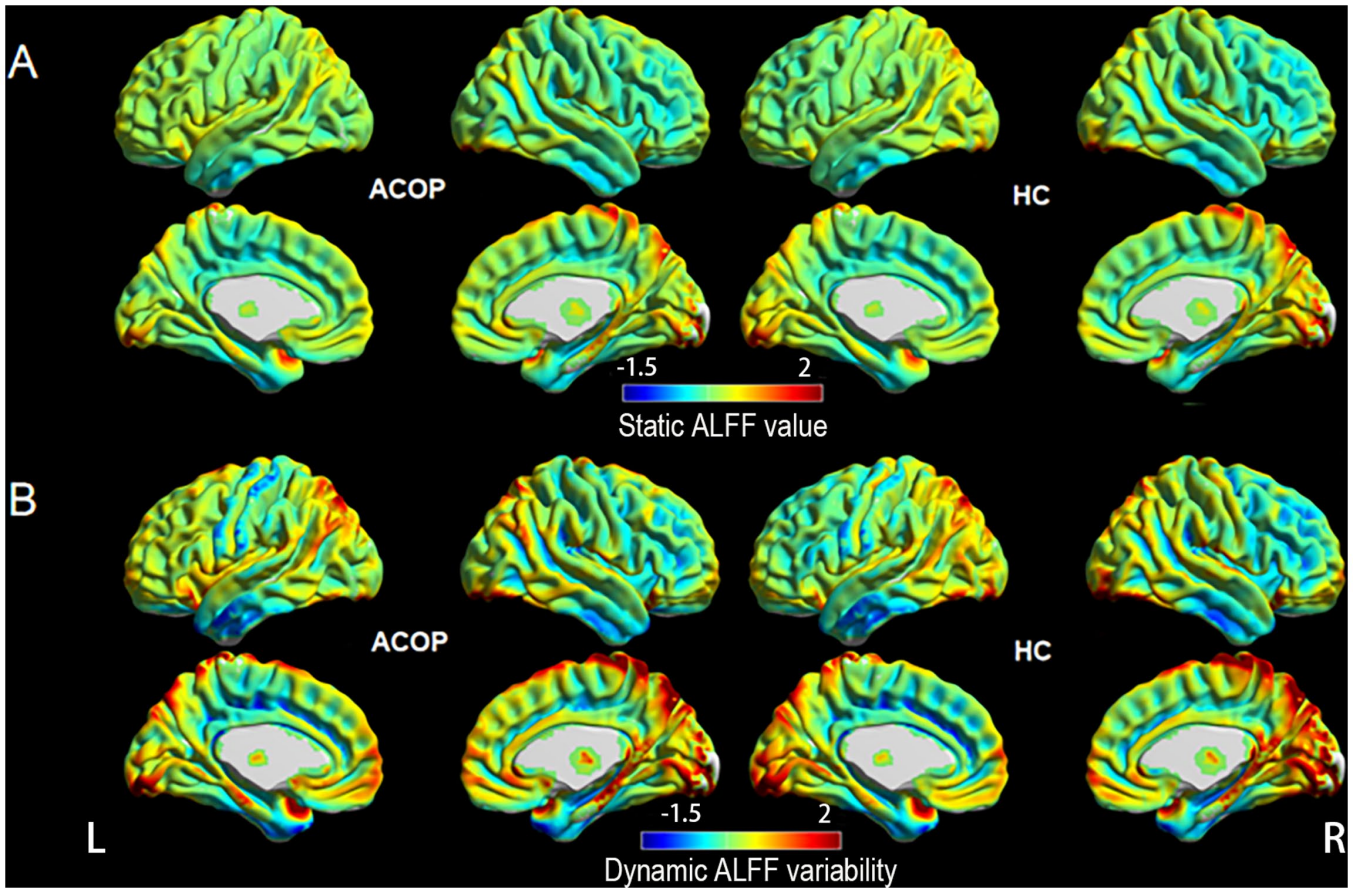
The distribution pattern of static ALFF value and dynamic ALFF variability in the ACOP patients and HC. **(A)** The distribution pattern of static ALFF value in the ACOP patients and HC. **(B)** The distribution pattern of dynamic ALFF variability in the ACOP patients and HC. ACOP, acute carbon monoxide poisoning; HC, healthy controls; ALFF, amplitude of low-frequency fluctuation; L, left; R, right.

**Figure 2:**
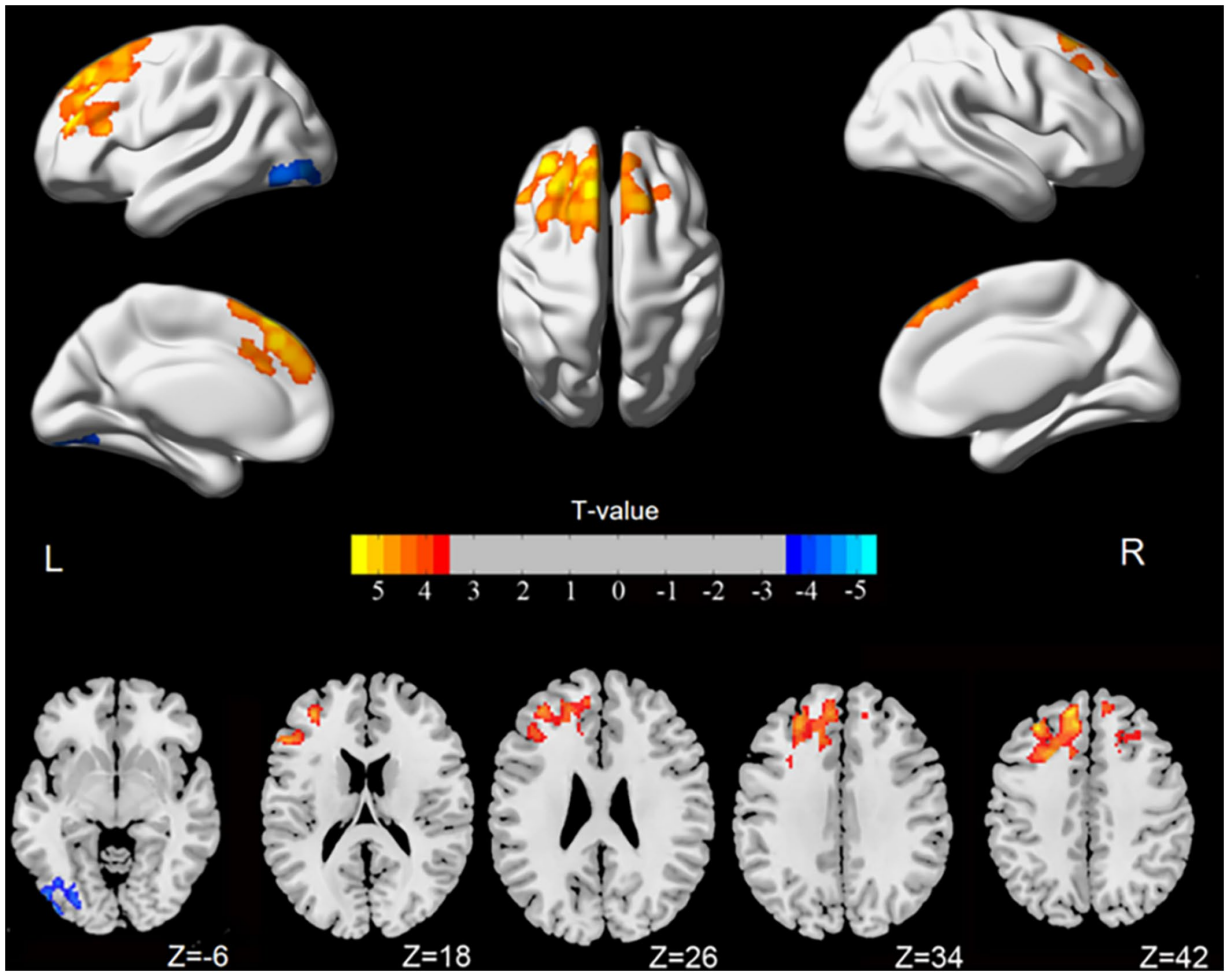
Brain areas showing significant differences in static ALFF comparing ACOP patients with HC [*p*-voxel < 0.001, *p*-cluster < 0.05, controlling for age, gender, education, and mean framewise displacement, Gaussian random field (GRF) correction]. Numbers in the color bars indicate *T*-values. ALFF, amplitude of low-frequency fluctuation; ACOP, acute carbon monoxide poisoning; HC, healthy controls; L, left; R, right.

**Figure 3:**
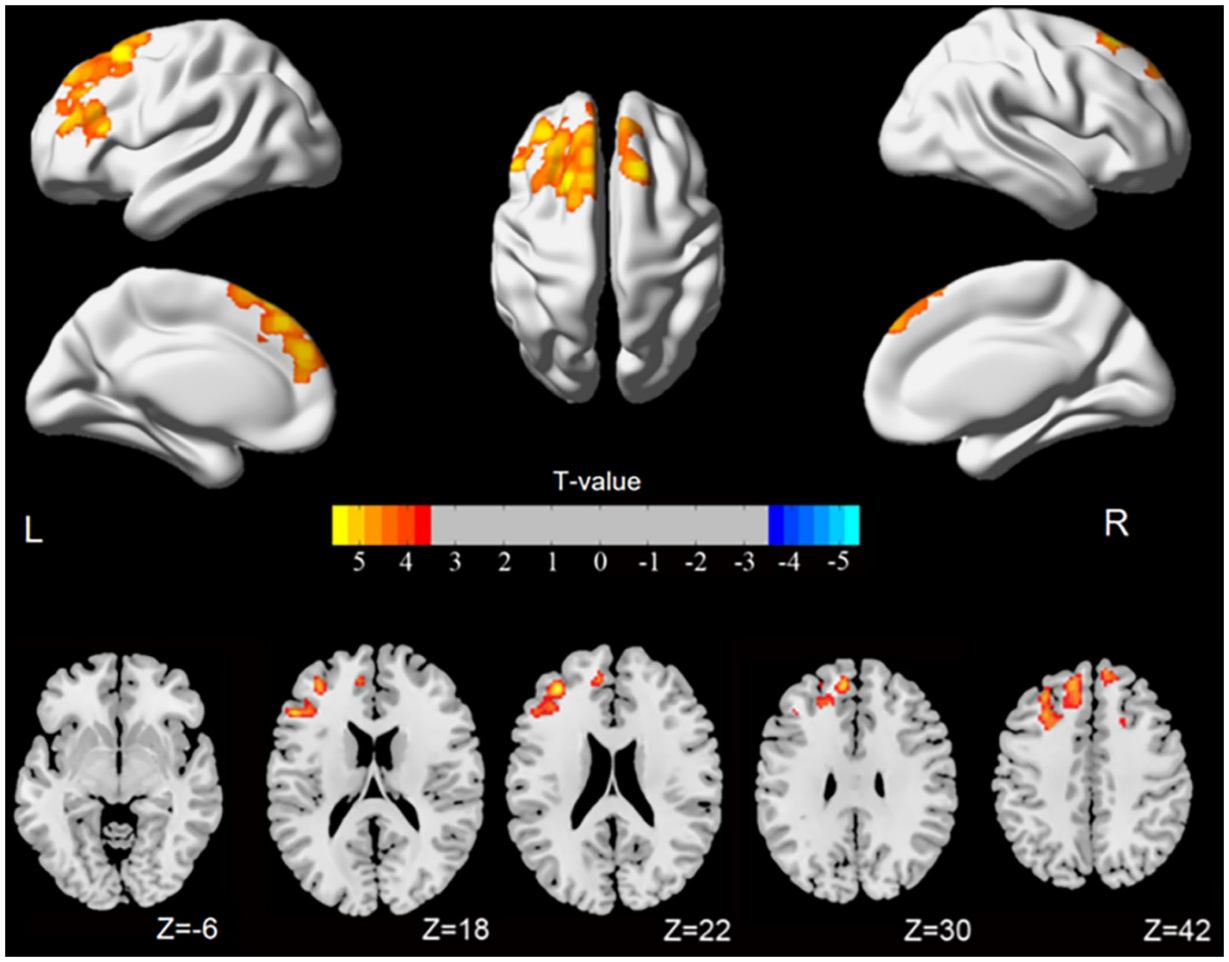
Brain areas showing significant differences in dynamic ALFF comparing ACOP patients with HC [*p*-voxel < 0.001, *p*-cluster < 0.05, controlling for age, gender, education, and mean framewise displacement, Gaussian random field (GRF) corrected]. The numbers in the color bars indicate *T*-values. ALFF, amplitude of low-frequency fluctuation; ACOP, acute carbon monoxide poisoning; HC, healthy controls; L, left; PAT, patients; R, right.

Figure 1 illustrates that the spatial variations in the static ALFF value and dynamic ALFF variability between the HC and ACOP groups were similar. Compared to the HC group, the ACOP group exhibited a reduced static ALFF in the left middle occipital gyrus (MOG), but an increased static ALFF in the left medial superior frontal gyrus (SFG) and right SFG (*p*-voxel < 0.001, *p*-cluster < 0.05, GRF corrected) (Table 2 and Figure 2). Similarly, individuals with ACOP exhibited increased dynamic ALFF variability in both the bilateral SFG and left middle frontal gyrus (MFG) compared to those in the HC group (*p-*voxel < 0.001, *p-*cluster < 0.05, GRF corrected) (Table 2 and Figure 3).

**Table 2:** Static ALFF value and dynamic ALFF variability in ACOP patients compared to HC.

Variables	Brain regions		Cluster size (voxels)	Peak MNI coordinates			*T*-values
	AAL	Brodmann		*x*	*y*	*z*	
Static ALFF	PAT > HC						
	Left medial superior frontal gyrus	BA8	2,543	−9.5	31.5	55.5	5.58
	Right superior frontal gyrus	BA8	428	14.5	23.5	59.5	4.79
	PAT < HC						
	Left middle occipital gyrus	BA19	428	−43.5	−84.5	−6.5	−4.42
Dynamic ALFF	PAT > HC						
	Left superior frontal gyrus	BA8	1,468	−23.5	17.5	57.5	5.53
	Right superior frontal gyrus	BA8	255	16.5	23.5	57.5	5.30
	Left middle frontal gyrus	BA46	368	−35.5	43.5	21.5	5.27

Statistical significance was set at *p*-voxel < 0.001 and *p*-cluster < 0.05, controlling for age, gender, education, mean framewise displacement, and Gaussian random field (GRF) correction. AAL, automated anatomical labeling; ALFF, the amplitude of low-frequency fluctuation; ACOP, acute carbon monoxide poisoning; HC, healthy controls; MNI, Montreal Neurological Institute; PAT, patients.

### Correlation analysis

**Figure 4:**
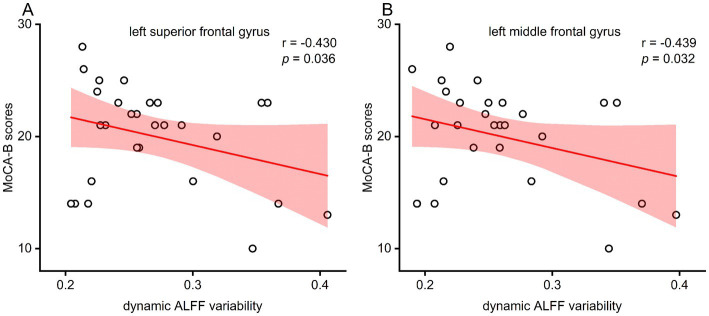
**(A)** Dynamic ALFF in the left superior frontal gyrus negatively correlated with the MoCA-B score in the ACOP group (*r* = −0.430, *p* = 0.036; corrected for age, gender, and education). **(B)** Dynamic ALFF in the left middle frontal gyrus negatively correlated with the MoCA-B score in the ACOP group (*r* = −0.439, *p* = 0.032; corrected for age, gender, and education). ALFF, amplitude of low-frequency fluctuation; MoCA-B, Montreal Cognitive Assessment Basic.

The results demonstrated that the aberrant dynamic ALFF in the left SFG and left MFG were inversely correlated with MoCA-B scores (*r* = −0.430, *p* = 0.036; *r* = −0.439, *p* = 0.032, respectively; see Figure 4). However, these findings did not support the Bonferroni correction. Furthermore, no significant correlations were observed among GCS scores, CO exposure duration, coma duration, and static/dynamic ALFF within the ACOP group.

### Validation analysis

Our main findings were supported by the results of sliding window lengths of 30 and 70 TR. Additionally, consistent results were observed when using a different increment of 4 TR, demonstrating similarity to the primary findings obtained with an increment of 1 TR in our study. The results of all validation analyses are detailed in the Supplementary Figures S1–S3.

## Discussion

The current study employed both static and dynamic ALFF methods to investigate temporal variations in the local intrinsic brain activity in patients with ACOP. In our study, patients with ACOP showed increased static ALFF value and dynamic ALFF variability in the bilateral SFG, increased dynamic ALFF variability in the left MFG, and decreased static ALFF in the left MOG. Furthermore, abnormal dynamic ALFF variability in the left SFG and left MFG was negatively correlated with cognitive performance in patients with ACOP.

The SFG is widely recognized as a crucial neural region involved in motor function (Martino et al., 2011), working memory (du Boisgueheneuc et al., 2006), and self-reflection, particularly in higher-order cognitive processing (Goldberg et al., 2006; Niendam et al., 2012; Briggs et al., 2020). It is noteworthy that memory impairment and cognitive dysfunction are common neurocognitive sequelae stemming from brain injury due to carbon monoxide poisoning (Ning et al., 2020). Our findings revealed that patients with ACOP exhibited increased static ALFF value and dynamic ALFF variability in the bilateral SFG. In a previous study on other types of acquired brain injury, it was found that patients with mild traumatic brain injury exhibited increased static ALFF values in the SFG. Concurrently, a network analysis using independent component analysis shows that functional connectivity is enhanced within the SFG (Palacios et al., 2013). Additionally, another study reported that increased functional connectivity of the medial SFG in patients with mild traumatic brain injury was negatively correlated with neuropsychological symptoms (Zhou et al., 2012). The medial SFG is a key hub of the default mode network (DMN) (Andrews-Hanna et al., 2014). The DMN is also a crucial component of cognitive function and is maintained by the brain to facilitate a state of readiness for incoming cognitive demands (Zidda et al., 2018). Furthermore, in our study, patients with ACOP displayed increased variability in the dynamic ALFF in the left MFG. The MFG, which encompasses the dorsolateral prefrontal cortex, is responsible for working memory, prospective memory, and executive functions (Barbey et al., 2013). This study’s observation of an increase in static ALFF value and dynamic ALFF variability within the SFG and MFG of patients with ACOP suggests unusual temporal fluctuations in local brain activity in these regions. These atypical patterns could potentially be linked to the cognitive impairments observed in ACOP.

In this study, both the static and dynamic ALFF analyses showed similar differences between the groups. However, only increased dynamic ALFF variability in the left SFG and left MFG of patients with ACOP showed a negative correlation with the MoCA-B scores. These findings suggest that individuals with more severe global cognitive impairment might exhibit more evident compensatory mechanisms in the temporal variability of local brain activity within the left SFG and MFG. Compensatory responses in intrinsic brain activity have also been observed in patients with traumatic brain injury (Palacios et al., 2013; Pagulayan et al., 2020). Alterations in temporal variability within the left SFG and left MFG may potentially act as biomarkers of cognitive states associated with ACOP. Nevertheless, because these correlations did not withstand correction for multiple comparisons, the correlation findings should be considered preliminary and may require validation using larger datasets.

A meta-analysis of the vulnerability of white matter in patients with CO poisoning found that, compared with the HC group, the white matter structure in most brain areas may be damaged after poisoning, but the data showed that the frontal-subcortical networks were the most vulnerable area (Hsiao et al., 2023). In our study, patients with ACOP exhibited abnormalities in static and dynamic temporal changes in the local intrinsic brain activity in the frontal lobe. This may be closely related to the development of brain tissue. The prefrontal subcortical region is where myelin forms last, leading to myelin in this area being much thinner than in other areas (Bartzokis, 2004). Children with a history of neonatal hypoxic–ischemic encephalopathy likewise show altered white matter connectivity within the frontal regions, including the superior and middle frontal gyri, associated with motor deficits (Spencer et al., 2021). Chronic high-altitude hypoxia has also been associated with reduced gray matter density and abnormal ALFF signals in the medial prefrontal areas (Zhang et al., 2022). These convergent findings suggest that the prefrontal and frontal-subcortical regions are consistently susceptible to different hypoxic, vascular, and metabolic insults, supporting the prefrontal abnormalities observed in ACOP. In a previous study on the topological properties of the brain’s structural network in patients with DEACMP, the nodal efficiency of the SFG in DEACMP was significantly decreased (Jiang et al., 2021). Furthermore, extensive metabolic analysis using ^18^F-fluorodeoxyglucose positron emission tomography and cognitive assessments has revealed that the network encompassing the frontal lobe, insula, and caudate nucleus constitutes the principal degenerative network in CO poisoning (Chang et al., 2011; Chen et al., 2015). Considering these findings, it has become increasingly evident that the frontal regions, particularly the prefrontal subcortical networks, may play a central role in the neuropathological consequences of CO poisoning. This may underscore the need for targeted therapeutic interventions and monitoring strategies for patients with ACOP to address potential frontal lobe dysfunction and mitigate long-term cognitive impairment. In particular, the association between abnormal dynamic ALFF variability in the SFG and MFG and global cognitive performance suggests that these prefrontal regions may serve as candidate targets for cognitive rehabilitation and neuromodulatory interventions aimed at enhancing executive and memory functions during recovery from CO poisoning. Moreover, dynamic ALFF may be explored as a functional biomarker to stratify patients for more intensive hyperbaric oxygen therapy and cognitive training programs and to monitor treatment response over time in future longitudinal studies.

Although our study revealed changes in spontaneous brain activity in patients with carbon monoxide poisoning, we acknowledge several limitations. First, the sample size of the patient group was relatively small, limiting the statistical power and ability to interpret robustly the potential relationship between brain activity and cognitive function. In future studies, we plan to increase our sample size and employ stricter inclusion criteria to minimize the variability among patients and address these concerns. Second, while abnormal static and dynamic ALFF were observed in patients with ACOP, these findings were based solely on a single modality of resting-state fMRI. The credibility of these results could be enhanced by incorporating multiple neuroimaging techniques, such as white matter fiber tracking, to investigate the structural mechanisms underlying altered ALFF. Finally, the present study is cross-sectional, and a longitudinal study would be more informative for revealing the functional changes in patients with ACOP during rehabilitation.

## Conclusion

This study provides novel insights into the alterations in both static and dynamic local intrinsic brain activities in patients with ACOP, with particular emphasis on the vulnerability of the prefrontal regions. Additionally, abnormal variability in dynamic ALFF correlated with cognitive function in patients with ACOP. Our results further illustrate that combining static and dynamic analyses may enhance our understanding of the neuropathological mechanisms driving brain disturbances resulting from CO exposure.

## Data Availability

The raw data supporting the conclusions of this article will be made available by the authors, without undue reservation.

## Footnotes

1
http://rfmri.org/DPABISurf

2
https://fmriprep.org

3
https://github.com/nipy/nipype

## References

[ref1] Aiello M. Salvatore E. Cachia A. Pappatà S. Cavaliere C. Prinster A. . (2015). Relationship between simultaneously acquired resting-state regional cerebral glucose metabolism and functional MRI: a PET/MR hybrid scanner study. NeuroImage 113, 111–121. doi: 10.1016/j.neuroimage.2015.03.017, 25791784

[ref2] Allen E. A. Damaraju E. Plis S. M. Erhardt E. B. Eichele T. Calhoun V. D. (2014). Tracking whole-brain connectivity dynamics in the resting state. Cereb. Cortex 24, 663–676. doi: 10.1093/cercor/bhs352, 23146964 PMC3920766

[ref3] Andrews-Hanna J. R. Smallwood J. Spreng R. N. (2014). The default network and self-generated thought: component processes, dynamic control, and clinical relevance. Ann. N. Y. Acad. Sci. 1316, 29–52. doi: 10.1111/nyas.12360, 24502540 PMC4039623

[ref4] Avants B. B. Epstein C. L. Grossman M. Gee J. C. (2008). Symmetric diffeomorphic image registration with cross-correlation: evaluating automated labeling of elderly and neurodegenerative brain. Med. Image Anal. 12, 26–41. doi: 10.1016/j.media.2007.06.004, 17659998 PMC2276735

[ref5] Barbey A. K. Koenigs M. Grafman J. (2013). Dorsolateral prefrontal contributions to human working memory. Cortex 49, 1195–1205. doi: 10.1016/j.cortex.2012.05.022, 22789779 PMC3495093

[ref6] Bartzokis G. (2004). Age-related myelin breakdown: a developmental model of cognitive decline and Alzheimer's disease. Neurobiol. Aging 25, 5–18; author reply 49-62. doi: 10.1016/j.neurobiolaging.2003.03.001, 14675724

[ref7] Bendell C. Moosavi S. H. Herigstad M. (2020). Low-level carbon monoxide exposure affects BOLD fMRI response. J. Cereb. Blood Flow Metab. 40, 2215–2224. doi: 10.1177/0271678X19887358, 31711340 PMC7585926

[ref8] Beppu T. (2013). The role of MR imaging in assessment of brain damage from carbon monoxide poisoning: a review of the literature. Am. J. Neuroradiol. 35, 625–631. doi: 10.3174/ajnr.A3489, 23598831 PMC7965807

[ref9] Briggs R. G. Khan A. B. Chakraborty A. R. Abraham C. J. Anderson C. D. Karas P. J. . (2020). Anatomy and white matter connections of the superior frontal gyrus. Clin. Anat. 33, 823–832. doi: 10.1002/ca.23523, 31749198

[ref10] Chang C.-C. Chang W.-N. Lui C.-C. Huang S.-H. Lee C.-C. Chen C. . (2011). Clinical significance of the pallidoreticular pathway in patients with carbon monoxide intoxication. Brain 134, 3632–3646. doi: 10.1093/brain/awr287, 22094539

[ref11] Chen N. C. Huang C. W. Huang S. H. Chang W. N. Chang Y. T. Lui C. C. . (2015). Cognitive severity-specific neuronal degenerative network in charcoal burning suicide-related carbon monoxide intoxication: a multimodality neuroimaging study in Taiwan. Medicine (Baltimore) 94:e783. doi: 10.1097/md.0000000000000783, 25984663 PMC4602570

[ref12] Chen W. Wang H. Sun T. Wu Q. Han W. Li Q. . (2022). Dynamic changes in fractional amplitude of low-frequency fluctuations in patients with chronic insomnia. Front. Neurosci. 16:1050240. doi: 10.3389/fnins.2022.1050240, 36523433 PMC9744813

[ref13] Cox R. W. Hyde J. S. (1997). Software tools for analysis and visualization of fMRI data. NMR Biomed. 10, 171–178. doi: 10.1002/(sici)1099-1492(199706/08)10:4/5<171::aid-nbm453>3.0.co;2-l, 9430344

[ref14] Cui Q. Sheng W. Chen Y. Pang Y. Lu F. Tang Q. . (2020). Dynamic changes of amplitude of low-frequency fluctuations in patients with generalized anxiety disorder. Hum. Brain Mapp. 41, 1667–1676. doi: 10.1002/hbm.24902, 31849148 PMC7267950

[ref15] Dale A. M. Fischl B. Sereno M. I. (1999). Cortical surface-based analysis. I. Segmentation and surface reconstruction. NeuroImage 9, 179–194. doi: 10.1006/nimg.1998.0395, 9931268

[ref16] Dinghua L. Dongbo L. Jianyu Z. Lan P. (2013). A resting-state functional magnetic resonance imaging study of acute carbon monoxide poisoning in humans. Cell Biochem. Biophys. 67, 1029–1032. doi: 10.1007/s12013-013-9600-1, 23553146

[ref17] Du Boisgueheneuc F. Levy R. Volle E. Seassau M. Duffau H. Kinkingnehun S. . (2006). Functions of the left superior frontal gyrus in humans: a lesion study. Brain 129, 3315–3328. doi: 10.1093/brain/awl244, 16984899

[ref18] Esteban O. Markiewicz C. J. Blair R. W. Moodie C. A. Isik A. I. Erramuzpe A. . (2019). fMRIPrep: a robust preprocessing pipeline for functional MRI. Nat. Methods 16, 111–116. doi: 10.1038/s41592-018-0235-4, 30532080 PMC6319393

[ref19] Fonov V. Evans A. C. Botteron K. Almli C. R. Mckinstry R. C. Collins D. L. (2011). Unbiased average age-appropriate atlases for pediatric studies. NeuroImage 54, 313–327. doi: 10.1016/j.neuroimage.2010.07.033, 20656036 PMC2962759

[ref20] Friston K. J. Williams S. Howard R. Frackowiak R. S. Turner R. (1996). Movement-related effects in fMRI time-series. Magn. Reson. Med. 35, 346–355. doi: 10.1002/mrm.1910350312, 8699946

[ref21] Fu Z. Tu Y. Di X. Du Y. Pearlson G. D. Turner J. A. . (2018). Characterizing dynamic amplitude of low-frequency fluctuation and its relationship with dynamic functional connectivity: an application to schizophrenia. NeuroImage 180, 619–631. doi: 10.1016/j.neuroimage.2017.09.035, 28939432 PMC5860934

[ref22] Goldberg I. Harel M. Malach R. (2006). When the brain loses its self: prefrontal inactivation during sensorimotor processing. Neuron 50, 329–339. doi: 10.1016/j.neuron.2006.03.015, 16630842

[ref23] Gorgolewski K. Burns C. D. Madison C. Clark D. Halchenko Y. O. Waskom M. L. . (2011). Nipype: a flexible, lightweight and extensible neuroimaging data processing framework in python. Front. Neuroinform. 5:13. doi: 10.3389/fninf.2011.00013, 21897815 PMC3159964

[ref24] Greve D. N. Fischl B. (2009). Accurate and robust brain image alignment using boundary-based registration. NeuroImage 48, 63–72. doi: 10.1016/j.neuroimage.2009.06.060, 19573611 PMC2733527

[ref25] Guo J. R. Shi J. Y. Dong Q. Y. Cao Y. B. Li D. Chen H. J. (2022). Altered dynamic spontaneous neural activity in minimal hepatic encephalopathy. Front. Neurol. 13:963551. doi: 10.3389/fneur.2022.963551, 36061995 PMC9439282

[ref26] Hampson N. B. Piantadosi C. A. Thom S. R. Weaver L. K. (2012). Practice recommendations in the diagnosis, management, and prevention of carbon monoxide poisoning. Am. J. Respir. Crit. Care Med. 186, 1095–1101. doi: 10.1164/rccm.201207-1284CI, 23087025

[ref27] Hoops S. Nazem S. Siderowf A. D. Duda J. E. Xie S. X. Stern M. B. . (2009). Validity of the MoCA and MMSE in the detection of MCI and dementia in Parkinson disease. Neurology 73, 1738–1745. doi: 10.1212/WNL.0b013e3181c34b47, 19933974 PMC2788810

[ref28] Hsiao W. C. Nouchi R. Chang H. I. Hsu S. W. Lee C. C. Huang S. H. . (2023). Clinical significance of fractional anisotropy in cerebral white matter regional vulnerability caused by carbon monoxide poisoning: a systematic review and meta-analysis. Neurotoxicology 96, 92–100. doi: 10.1016/j.neuro.2023.04.005, 37060949

[ref29] Jenkinson M. Bannister P. Brady M. Smith S. (2002). Improved optimization for the robust and accurate linear registration and motion correction of brain images. NeuroImage 17, 825–841. doi: 10.1016/s1053-8119(02)91132-8, 12377157

[ref30] Jiang W. Zhao Z. Wu Q. Wang L. Zhou L. Li D. . (2021). Study on brain structure network of patients with delayed encephalopathy after carbon monoxide poisoning: based on diffusion tensor imaging. Radiol. Med. 126, 133–141. doi: 10.1007/s11547-020-01222-x, 32557108

[ref31] Klein A. Ghosh S. S. Bao F. S. Giard J. Häme Y. Stavsky E. . (2017). Mindboggling morphometry of human brains. PLoS Comput. Biol. 13:e1005350. doi: 10.1371/journal.pcbi.1005350, 28231282 PMC5322885

[ref32] Leonardi N. Van De Ville D. (2015). On spurious and real fluctuations of dynamic functional connectivity during rest. NeuroImage 104, 430–436. doi: 10.1016/j.neuroimage.2014.09.007, 25234118

[ref33] Liao W. Li J. Ji G. J. Wu G. R. Long Z. Xu Q. . (2019). Endless fluctuations: temporal dynamics of the amplitude of low frequency fluctuations. IEEE Trans. Med. Imaging 38, 2523–2532. doi: 10.1109/tmi.2019.2904555, 30872224

[ref34] Liao W. Wang J. Xu T. Zhang Z. Ji G.-J. Xu Q. . (2016). Altered relationship between thickness and intrinsic activity amplitude in generalized tonic–clonic seizures. Sci. Bull. 61, 1865–1875. doi: 10.1007/s11434-016-1201-0

[ref35] Liu J. Bu X. Hu X. Li H. Cao L. Gao Y. . (2021). Temporal variability of regional intrinsic neural activity in drug-naïve patients with obsessive-compulsive disorder. Hum. Brain Mapp. 42, 3792–3803. doi: 10.1002/hbm.25465, 33949731 PMC8288087

[ref36] Lo C.-P. Chen S.-Y. Lee K.-W. Chen W.-L. Chen C.-Y. Hsueh C.-J. . (2007). Brain injury after acute carbon monoxide poisoning: early and late complications. Am. J. Roentgenol. 189, W205–W211. doi: 10.2214/AJR.07.2425, 17885032

[ref37] Martino J. Gabarrós A. Deus J. Juncadella M. Acebes J. J. Torres A. . (2011). Intrasurgical mapping of complex motor function in the superior frontal gyrus. Neuroscience 179, 131–142. doi: 10.1016/j.neuroscience.2011.01.047, 21277357

[ref38] Mattiuzzi C. Lippi G. (2020). Worldwide epidemiology of carbon monoxide poisoning. Hum. Exp. Toxicol. 39, 387–392. doi: 10.1177/0960327119891214, 31789062

[ref39] Medaglia J. D. (2017). Functional neuroimaging in traumatic brain injury: from nodes to networks. Front. Neurol. 8:407. doi: 10.3389/fneur.2017.00407, 28883806 PMC5574370

[ref40] Niendam T. A. Laird A. R. Ray K. L. Dean Y. M. Glahn D. C. Carter C. S. (2012). Meta-analytic evidence for a superordinate cognitive control network subserving diverse executive functions. Cogn. Affect. Behav. Neurosci. 12, 241–268. doi: 10.3758/s13415-011-0083-5, 22282036 PMC3660731

[ref41] Ning K. Zhou Y. Y. Zhang N. Sun X. J. Liu W. W. Han C. H. (2020). Neurocognitive sequelae after carbon monoxide poisoning and hyperbaric oxygen therapy. Med. Gas Res. 10, 30–36. doi: 10.4103/2045-9912.279981, 32189667 PMC7871936

[ref42] Pagulayan K. F. Petrie E. C. Cook D. G. Hendrickson R. C. Rau H. Reilly M. . (2020). Effect of blast-related mTBI on the working memory system: a resting state fMRI study. Brain Imaging Behav. 14, 949–960. doi: 10.1007/s11682-018-9987-9, 30519997 PMC11835419

[ref43] Palacios E. M. Sala-Llonch R. Junque C. Roig T. Tormos J. M. Bargallo N. . (2013). Resting-state functional magnetic resonance imaging activity and connectivity and cognitive outcome in traumatic brain injury. JAMA Neurol. 70, 845–851. doi: 10.1001/jamaneurol.2013.38, 23689958

[ref44] Park H. J. Friston K. J. Pae C. Park B. Razi A. (2018). Dynamic effective connectivity in resting state fMRI. NeuroImage 180, 594–608. doi: 10.1016/j.neuroimage.2017.11.033, 29158202 PMC6138953

[ref45] Rose J. J. Wang L. Xu Q. Mctiernan C. F. Shiva S. Tejero J. . (2017). Carbon monoxide poisoning: pathogenesis, management, and future directions of therapy. Am. J. Respir. Crit. Care Med. 195, 596–606. doi: 10.1164/rccm.201606-1275CI, 27753502 PMC5363978

[ref46] Spencer A. P. C. Brooks J. C. W. Masuda N. Byrne H. Lee-Kelland R. Jary S. . (2021). Motor function and white matter connectivity in children cooled for neonatal encephalopathy. Neuroimage Clin. 32:102872. doi: 10.1016/j.nicl.2021.102872, 34749285 PMC8578038

[ref47] Tustison N. J. Avants B. B. Cook P. A. Zheng Y. Egan A. Yushkevich P. A. . (2010). N4ITK: improved N3 bias correction. IEEE Trans. Med. Imaging 29, 1310–1320. doi: 10.1109/tmi.2010.2046908, 20378467 PMC3071855

[ref48] Weaver L. K. (2009). Clinical practice. Carbon monoxide poisoning. N. Engl. J. Med. 360, 1217–1225. doi: 10.1056/NEJMcp0808891, 19297574

[ref49] Wu K. Liu M. Zhao G. He L. Tan Y. (2020). Altered regional homogeneity in delayed encephalopathy after carbon monoxide poisoning: a resting-state fMRI study. Neurosci. Lett. 729:135002. doi: 10.1016/j.neulet.2020.135002, 32334106

[ref50] Yan C. G. Wang X. D. Zuo X. N. Zang Y. F. (2016). DPABI: data processing & analysis for (resting-state) brain imaging. Neuroinformatics 14, 339–351. doi: 10.1007/s12021-016-9299-4, 27075850

[ref51] Zang Y. F. He Y. Zhu C. Z. Cao Q. J. Sui M. Q. Liang M. . (2007). Altered baseline brain activity in children with ADHD revealed by resting-state functional MRI. Brain Dev. 29, 83–91. doi: 10.1016/j.braindev.2006.07.002, 16919409

[ref52] Zhang Y. Brady M. Smith S. (2001). Segmentation of brain MR images through a hidden Markov random field model and the expectation-maximization algorithm. IEEE Trans. Med. Imaging 20, 45–57. doi: 10.1109/42.906424, 11293691

[ref53] Zhang Y. Q. Zhang W. J. Liu J. H. Ji W. Z. (2022). Effects of chronic hypoxic environment on cognitive function and neuroimaging measures in a high-altitude population. Front. Aging Neurosci. 14:788322. doi: 10.3389/fnagi.2022.788322, 35601614 PMC9122256

[ref54] Zhou Y. Milham M. P. Lui Y. W. Miles L. Reaume J. Sodickson D. K. . (2012). Default-mode network disruption in mild traumatic brain injury. Radiology 265, 882–892. doi: 10.1148/radiol.12120748, 23175546 PMC3504316

[ref55] Zidda F. Andoh J. Pohlack S. Winkelmann T. Dinu-Biringer R. Cavalli J. . (2018). Default mode network connectivity of fear- and anxiety-related cue and context conditioning. NeuroImage 165, 190–199. doi: 10.1016/j.neuroimage.2017.10.024, 29050910

