# Supplementary material for: Altered static and dynamic amplitude of low-frequency fluctuations in acute carbon monoxide poisoning patients: a resting-state fMRI study
Source: Front Neurosci. 2026 Jan 13;19:1695556. doi: 10.3389/fnins.2025.1695556 (PMC12835238; doi:10.3389/fnins.2025.1695556)
Supplement: Supplementary file 1 [file Data_Sheet_1.pdf]

Supplementary Material

We applied 50 TRs as window length, 1 TR as step size, 6 mm as smoothing core in our main research. To test if the different window length and step size have effects on our results, we tried other parameter, and the figure below demonstrated the stability of our experiments.

1 Results from different window length

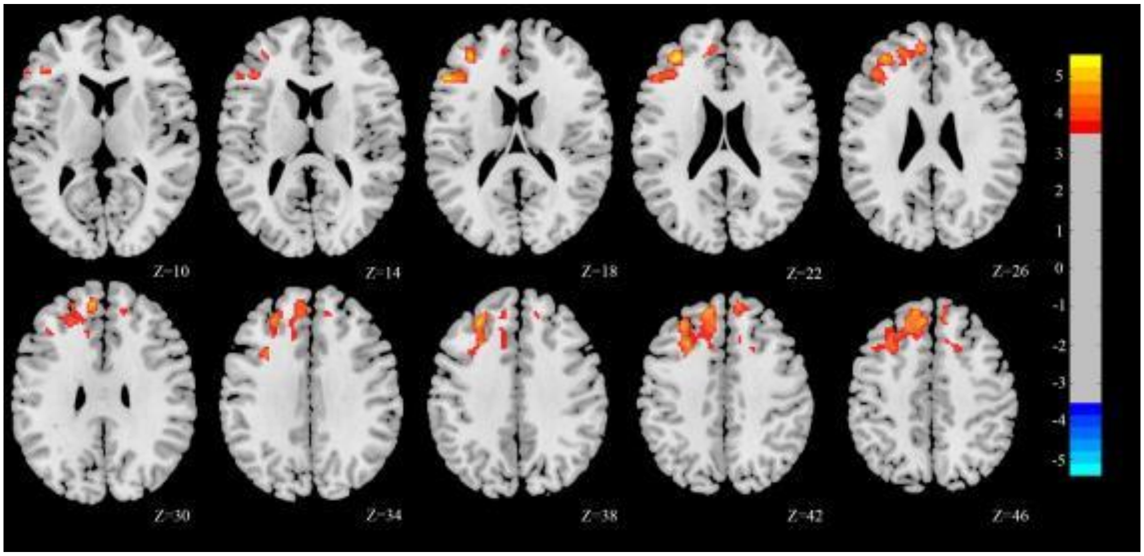

Supplementary Figure 1. Brain areas that show significant differences in dynamic ALFF (Window length, 30 TRs; Window step size, 1 TR; Gaussian smooth kernel, 6 mm) when comparing ACOP patients with HC [p-voxel < 0.001, p-cluster < 0.05, controlling for age, gender, education, and mean framewise displacement, Gaussian random field (GRF) corrected]. The numbers in the color bars indicate T-values. Individuals with ACOP still exhibited increased dynamic ALFF variability in both the bilateral superior frontal gyrus and left middle frontal gyrus compared to those in the HC group. ALFF, amplitude of low-frequency fluctuation; ACOP, acute carbon monoxide poisoning; HC, healthy controls

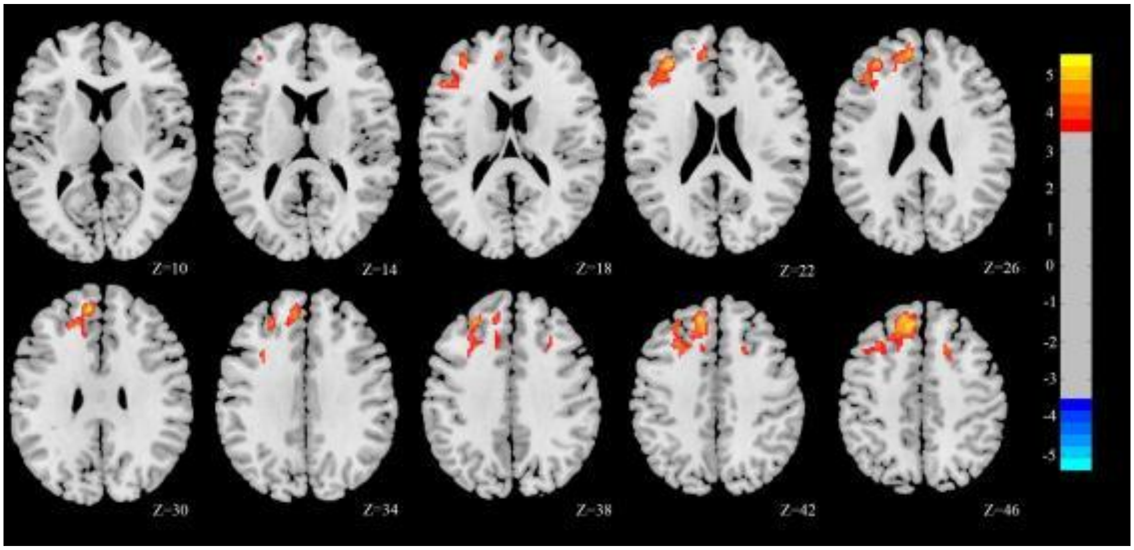

Supplementary Figure 2. Brain areas that show significant differences in dynamic ALFF (Window length, 70 TRs; Window step size, 1 TR; Gaussian smooth kernel, 6 mm) when comparing ACOP patients with HC [p-voxel < 0.001, p-cluster < 0.05, controlling for age, gender, education, and mean framewise displacement, Gaussian random field (GRF) corrected]. The numbers in the color bars indicate T-values. Individuals with ACOP still exhibited increased dynamic ALFF variability in both the bilateral superior frontal gyrus and left middle frontal gyrus compared to those in the HC group. ALFF, amplitude of low-frequency fluctuation; ACOP, acute carbon monoxide poisoning; HC, healthy controls

2 Results from different window step size

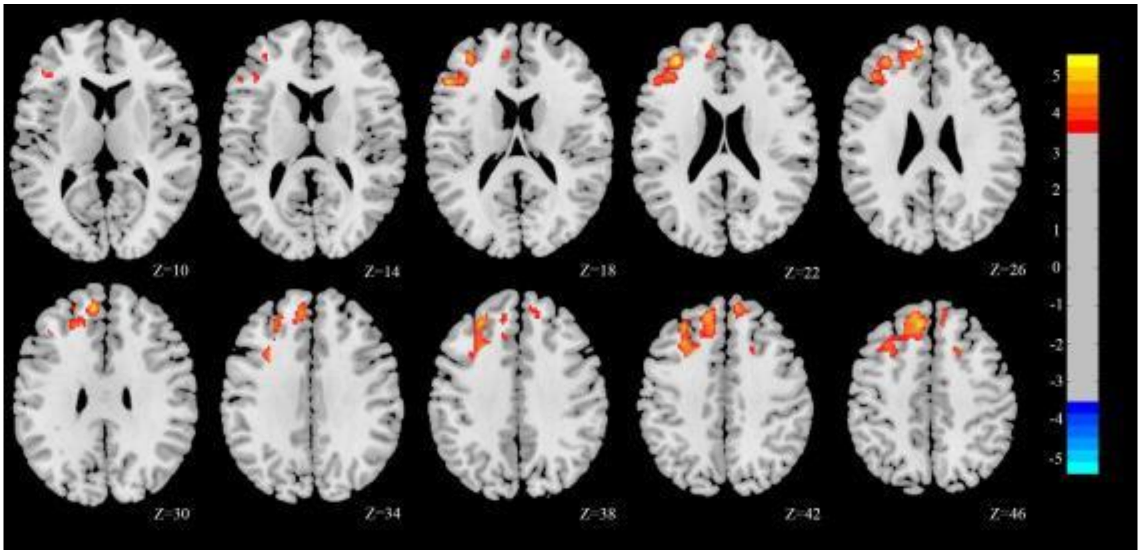

Supplementary Figure 3. Brain areas that show significant differences in dynamic ALFF (Window length, 50 TRs; Window step size, 4 TRs; Gaussian smooth kernel, 6 mm) when comparing ACOP patients with HC [p-voxel < 0.001, p-cluster < 0.05, controlling for age, gender, education, and mean framewise displacement, Gaussian random field (GRF) corrected]. The numbers in the color bars indicate T-values. Individuals with ACOP still exhibited increased dynamic ALFF variability in both the bilateral superior frontal gyrus and left middle frontal gyrus compared to those in the HC group. ALFF, amplitude of low-frequency fluctuation; ACOP, acute carbon monoxide poisoning; HC, healthy controls
